# Could polymorphisms of some hormonal receptor genes, involved in folliculogenesis help in predicting patient response to controlled ovarian stimulation?

**DOI:** 10.1007/s10815-018-1357-4

**Published:** 2018-11-08

**Authors:** Maruška Čuš, Veljko Vlaisavljević, Katja Repnik, Uroš Potočnik, Borut Kovačič

**Affiliations:** 1 Department of Reproductive Medicine and Gynecological Endocrinology, University Medical Centre Maribor, Ljubljanska ulica 5, 2000 Maribor, Slovenia; 2 Biomedical Research Institute BRIS, Ljubljana, Slovenia; 3 Center for human molecular genetics and pharmacogenomics, Faculty of Medicine, University of Maribor, Maribor, Slovenia; 4 Faculty for Chemistry & Chemical Engineering, University of Maribor, Smetanova 17, Maribor, Slovenia

**Keywords:** Controlled ovarian hyperstimulation, Single nucleotide polymorphisms, FSHR, AMHR, Genotyping

## Abstract

**Purpose:**

The aim of this study was to investigate whether single nucleotide polymorphisms (SNPs) in selected genes, responsible for hormonal regulation of folliculogenesis, are associated with response to controlled ovarian hyperstimulation (COH) and clinical characteristics of women enrolled in in vitro fertilization (IVF) programs.

**Methods:**

In a cross-sectional study, 60 (IVF) patients underwent COH by using gonadotropin-releasing hormone (GnRH) antagonist and recombinant follicle-stimulating hormone (rFSH) protocol. Patients were classified into three groups: poor-responders (according to Bologna criteria), normo-responders (≤ 15 oocytes), and hyper-responders (> 15 oocytes). Genotyping of SNPs *AMH* rs10407022, *AMHR* rs3741664, *FSHR* rs1394205 and rs6166, and *ESR1* rs2234693 was performed using high-resolution melting analysis (HRMA). Basal FSH (bFSH), estradiol (E2), and anti-Müllerian hormone (AMH) were measured by enzyme-linked immunosorbent assay (ELISA).

**Results:**

Patients with GG genotype of *FSHR* rs1394205 had significantly lower AMH level (*P* = 0.016) and required higher rFSH dose per oocyte compared to women with AA or AG genotype (*P* = 0.036). We also found higher frequency of GG genotype of *FSHR* rs1394205 in poor- (76.5%) than in hyper-responders (37.5%, *P* = 0.002). Patients with AA genotype of *FSHR* rs6166 had higher level of measured bFSH compared to those with AG or GG genotypes (*P* = 0.043). Women with GG genotype of *AMHR* rs3741664 required higher rFSH dose in comparison with patients carrying genotypes AA or AG (*P* = 0.028).

**Conclusions:**

The GG genotype at position rs1394205 is associated with poor ovarian response to COH. Patients with this genotype may require higher doses of rFSH for ovulation induction.

## Introduction

Since introduction of IVF in the clinical practice of infertility treatment, the follicular response to ovarian stimulation protocols has been largely investigated. The unpredictable ovarian response to gonadotropins among patients, ranging from poor response to ovarian hyperstimulation syndrome (OHSS) has been one of the most challenging problems in medically assisted reproduction (MAR) [1]. The ovarian response to gonadotrophin stimulation is difficult to predict even in patients with similar endocrine profiles. This has led to the investigation of specific new biomarkers that could serve as predictors of ovarian response to an exogenous hormonal stimulation. Recently, gene association studies have tried to identify a number of genetic variations influencing inter-individual variability in COH [2, 3].

Since the first report on the polymorphism of the *FSHR* gene [4], numerous mutations and SNPs of the *FSHR* gene have been described. Very common coding SNPs rs6165, rs6166, and rs1394205 are currently most extensively studied to assess the response of the FSHR protein to FSH stimulation. Some authors have reported predictability of the ovarian response to FSH stimulation in patients with different alleles [5–10], while others have refuted this finding [11, 12]. The results of studies regarding the impact of ethnicity on the frequency distribution of follicle-stimulating hormone receptor (FSHR) polymorphisms are contradictory. Some studies mention the influence of ethnicity on the distribution of the genotype [13], while others contradict it [8].

Important candidate genes involved in the ovarian response to exogenous FSH are the estrogen receptor genes (*ESRs*) [14]. The most studied polymorphisms in *ESR1* are rs2234693 and rs9340799. Women with TT genotype at rs2234693 when compared to those with CC genotype, demonstrate improved quality of the ovarian follicles [15], as well as higher number of follicles, mature oocytes, higher fertilization rate, and better embryo quality following COH and IVF [16–18]. The results of meta-analysis done on Asian population strongly suggested that *ESR1* gene rs2234693 polymorphism was significantly associated with an increased risk of premature ovarian failure [19].

Rigon et al. [20] confirmed an association of the *AMH* gene polymorphisms, and its receptor *AMHR* with estradiol levels during the follicular phase of the menstrual cycle in normo-ovulatory women. AMH is produced by the granulosa cells of early developing follicles in the ovary and it continues to be expressed in the growing follicles until they are selected for dominance by the action of follicle-stimulating hormone (FSH) [21, 22]. Studies in *AMH* knockout mice have demonstrated that, in the absence of *AMH*, follicles are recruited at a faster rate, and they are more sensitive to FSH [23], suggesting that serum AMH could inhibit primordial follicle development and be induced by FSH. This expression pattern suggests that *AMH* can inhibit both the initiation of primordial follicle growth and FSH-induced follicle growth. Therefore, *AMH* plays an important role in regulating both primordial follicle recruitment and cyclic selection [24, 25]. Because AMH may have an inhibitory effect on the FSH-sensitivity of follicles, polymorphisms on the *AMH* gene or its receptor *AMHR* might reduce the biological activity of the hormone. Therefore, follicles might be more sensitive to FSH and might be previously selected for dominance [26].

The aim of our study was to analyze SNPs in selected candidate genes which are responsible for the hormonal regulation of folliculogenesis and to determine whether the reasons for the different ovarian responses to COH derive from a difference in individual genotype.

## Materials and methods

### Patients and ovarian stimulation

Sixty (60) women undergoing ovarian stimulation between March 1, 2015 and July 7, 2015 were included in this study. The study was approved by the Slovenian National Committee for Medical Ethics (012-347/2015-8). It is a part of the research program P3-0327 and the research project J3-7177 funded by the Slovenian Research Agency. Patients were included in the study after signing a written consent.

Patients were classified into three groups according to the ovarian response to stimulation protocol and the number of oocytes obtained after oocyte pick up. Group 1 were patients with a poor ovarian response (POR) and with up to three oocytes obtained and with additional risk factors for POR: antral follicle count < 5 or AMH < 0.5 ng/mL [23]. Group 2 represented women with normal response—those with up to 15 oocytes and Group 3 of hyper responders with more than 15 oocytes.

Exclusion criteria were endometriosis and polycystic ovary syndrome and age of more than 39 years.

Of the 60 included women, 26 (43.4%) were classified as normal responders, 17 (28.3%) hyper responders, and 17 (28.3%) as poor ovarian responders.

Blood samples were taken for basal hormonal measurements performed by enzyme-linked immunosorbent assay (ELISA). Serum FSH level was measured on day 3 of the menstrual cycle. Serum E2 level was measured on the day of hCG administration. Serum AMH level was determined independently of the menstrual cycle.

### Ovarian hyperstimulation and oocyte pick-up

Patients were assigned to ovarian stimulation with a combination of GnRH antagonist cetrorelix (Cetrotide 0.25 mg®; Merck Serono, Switzerland) and recombinant FSH (Gonal-F®, Merck Serono, Switzerland). The dose of exogenous gonadotropins was adjusted according to the follicular response, followed by serial transvaginal ultrasonography and E_2_ level measurement. When the follicle had reached 18 mm in diameter, 250 μg recombinant human chorionic gonadotropin (Ovitrelle ®; Merck Serono, Switzerland) was given subcutaneously to induce ovulation. Oocyte retrieval was performed by ultrasound-guided aspiration of follicles, 35 to 36 h after recombinant human chorionic gonadotropin administration. The cumulus-oocyte complexes from follicular aspirates were collected in oocyte collection medium and incubated in a CO_2_ incubator before insemination.

### DNA samples

DNA samples were obtained from 9 mL of the patient’s peripheral blood. First peripheral blood lymphocytes were collected using FicollPaque PLUS (GE Healthcare, Uppsala, Sweden) and then DNA was isolated from lymphocytes using TRI reagent (Sigma, Steinheim, Germany) according to the manufacturer’s instructions.

Genotyping of SNPs rs10407022 in gene *AMH*, rs3741664 in gene *AMHR*, rs1394205 and rs6166 in gene *FSHR*, and rs2234693 in gene *ESR1* was performed using polymerase chain reactions (PCR) followed by high-resolution melting analysis (HRMA). Forward and reverse primer sequences, primer concentrations, and annealing temperatures are shown in Table 1. Genotyping was performed on real time PCR LC480 instrument (Roche, Germany), using LC480 HRM Master Mix (Roche, Germany). Conditions were as follow: initial denaturation at 95 °C for 10 min, followed by 45 cycles of 95 °C for 10 s, 60 °C for 15 s and 72 °C for10 s, followed by HRM step of 95 °C for 1 min, 40 °C for 1 min, and 60–90 °C at 0.02 °C/s. Genotypes were determined using gene-scanning analysis software.

**Table 1:** Forward and reverse primer sequences, primer concentrations, and annealing temperatures

Gene	SNP ID	Variation	Region	Forward and reverse primer	Annealing temperature (°C)	Primer concentration (nM)	Genotyping method
*FSHR*	rs1394205	-29G/A	Non-coding	AGCTTCTGAGATCTGTGGAGG	62	300	HRM
				AGCAAAGAGACCAGGAGCAG			
	rs6166	Asn680Ser	Coding	CTTCAGCTCCCAGAGTCACC	62	300	HRM
				CATTGTGTTTTAGTTTTGGGCTAA			
*AMHR*	rs3741664	4952G/A	Non-coding	CGTCTCCAGCTTTGTGTACC	62	400	HRM
				GTCACTGGTGTACTGGGTCA			
*ESR1*	rs2234693	PvuII T/C	Coding	TGTTCTGTGTTGTCCATCAGT	62	400	HRM
				CTCTAGACCACACTCAGGGT			
*AMH*	rs10407022	Ile49Ser	Coding	TCCGAGAAGACTTGGACTGG	62	300	HRM
				AGCTGCTGCCATTGCTGT			

Notes

*HRM* high resolution melting

### Statistical analysis

T-test or Mann Whitney U-test was used to assess the statistical differences between groups of patients and biological and clinical parameters. To compare genotype and allele frequencies of selected SNPs between groups of patients, two-sided Fischer’s exact test was used. The data obtained were presented as mean ± standard deviation (SD). *P* < 0.05 was considered statistically significant. Odds ratios (OR) were also calculated with 95% confidence interval (95% CI).

## Results

Clinical and biological characteristics of patients, classified in three groups according to the ovarian response to gonadotropin are shown in Table 2. Results showed significant difference in age between groups. Mean age of poor responding patients was higher (34.9 years) in comparison with normo- (32.5 years, *P* = 0.019) and hyper-responders (30.8 years, *P* < 0.0005). Level of serum FSH was significantly higher in the group of poor responders (9.42 mIU/mL) compared to normo- (5.59 mIU/mL *P* = 0.046) and hyper-responders (5.55 mIU/mL, *P* = 0.025). Serum AMH level was significantly different between all groups (*P* < 0.05). Number of aspirated follicles, number of retrieved oocytes, and dosage of rFSH per oocyte retrieved were significantly different between groups (*P* < 0.05). Applicated rFSH dose was significantly higher in the group of poor responders, compared to normo- (*P* = 0.002) and hyper-responders (*P* < 0.0005).

**Table 2:** Main characteristic of study participants and analyzed parameters

	All participants	Poor responders	Normo responders	Hyper responders	*P* value		
					Poor vs. normo	Normo vs. hyper	Poor vs hyper
No.	60	17	26	17			
Age (years)	32.72 ± 0.66	34.92 ± 0.76	32.50 ± 1.15	30.85 ± 1.11	**0.019**	0.067	**< 0.0005**
BMI (kg/m^2^)	24.72 ± 0.82	23.76 ± 1.17	25.90 ± 1.32	23.87 ± 1.71	0.808	0.475	0.648
bFSH (mIU/mL)	6.66 ± 0.49	9.42 ± 1.37	5.59 ± 0.31	5.55 ± 0.43	**0.046**	0.322	**0.025**
AMH (ng/mL)	3.55 ± 0.54	0.46 ± 0.10	3.94 ± 0.80	6.05 ± 0.96	**< 0.0005**	**0.011**	**< 0.0005**
Estradiol on hCG day (pmol/L)	4.99 ± 0.62	2.82 ± 0.81	3.96 ± 0.57	8.75 ± 1.41	0.078	**0.005**	**< 0.0005**
No. of follicles punctured	12.44 ± 1.40	3.77 ± 1.00	10.57 ± 1.17	23.69 ± 2.19	**< 0.0005**	**< 0.0005**	**< 0.0005**
Oocytes retrieved	10.72 ± 1.16	3.54 ± 0.94	8.90 ± 0.70	20.69 ± 1.65	**< 0.0005**	**< 0.0005**	**< 0.0005**
rFSH (IU)	1855.44 ± 127.83	2688.46 ± 299.56	1631.25 ± 91.35	1367.31 ± 150.99	**0.002**	0.056	**< 0.0005**
rFSH (IU) per oocyte	462.99 ± 115.84	1233.52 ± 325.65	217.56 ± 27.60	70.05 ± 10.38	**< 0.0005**	**< 0.0005**	**0.001**

Notes: *P* < 0.05 was considered statistically significant. Statistically significant values are written in bold

BMI body mass index, bFSH basal follicle-stimulating hormone, AMH basal anti-Müllerian hormone, hCG human chorionic gonadotropin, rFSH recombinant follicle-stimulating hormone, IU international units

*P* is from t-test

Genotype and allele frequencies were calculated for patients as a whole group and separately according to response on COH. When comparing genotype and allele frequencies between groups, we found higher frequency of GG genotype of SNP rs1394205 in *FSHR* gene in poor- than in hyper-responders (76.5 vs. 37.5%, *P* = 0.002). The G allele was present in higher frequency in the group of poor responders compared to normo-responders (88.2 vs. 53.8%, *P* = 0.001, OR = 0.156) and hyper responders (88.2 vs. 62.5%, *P* = 0.015). Distribution of analyzed SNP genotypes of other genes was not significantly different between three patient groups (Table 3).

**Table 3:** Associations between selected SNPs and response to hormonal regulated folliculogenesis

Gene/SNP ID	Genotype/allele	All participants	Poor responders	Normo responders		Hyper responders
*AMH* rs10407022		*N* = 58	*N* = 17	*N* = 25		*N* = 16
	TT	(*n* = 42) 72.4%	(*n* = 13) 76.5%	(*n* = 18) 72%		(*n* = 11) 68.8%
	GT	(*n* = 14) 24.2%	(*n* = 4) 23.5%	(*n* = 5) 20%		(*n* = 5) 31.2%
	GG	(*n* = 2) 3.4%	(*n* = 0) 0%	(*n* = 2) 8%		(*n* = 0) 0%
	T	0.845	0.882	0.820		0.844
	G	0.155	0.118	0.180		0.156
	Statistical analysis	Poor vs. normo	Normo vs. hyper	Poor vs. hyper		TT vs. GT+GG
		1.000	0.723	0.708	*P* value	
		1.026	1.439	1.477	OR	
		0.241–4.369	0.355–5.837	0.317–6.895	95% CI	
		0.586	0.946	0.648	*P* value	T vs. G
		1.429	0.972	1.389	OR	
		0.394–5.182	0.288–3.285	0.338–5.711	95% CI	
*AMHR* rs1741664		*N* = 57	*N* = 17	*N* = 25		*N* = 15
	AA	(*n* = 0) 0%	(*n* = 0) 0%	(*n* = 0) 0%		(*n* = 0) 0%
	AG	(*n* = 18) 31.6%	(*n* = 3) 17.6%	(*n* = 10) 40%		(*n* = 5) 33.3%
	GG	(*n* = 39) 68.4%	(*n* = 14) 82.4%	(*n* = 15) 60%		(*n* = 10) 66.7%
	**A**	0.158	0.088	0.200		0.167
	**G**	0.842	0.912	0.800		0.833
	Statistical analysis	Poor vs. normo	Normo vs. hyper	Poor vs. hyper		
		0.190	0.729	0.671	*P* value	AG vs. GG
		0.321	1.667	0.536	OR	
		0.073–1.414	0.407–6.818	0.098–2.941	95% CI	
		0.181	0.528	0.526	*P* value	A vs. G
		0.400	1.500	0.600	OR	
		0.101–1.581	0.423–5.315	0.122–2.943	95% CI	
*FSHR* rs1394205		*N* = 59	*N* = 17	*N* = 26		*N* = 16
	AA	(*n* = 7) 11.9%	(*n* = 0) 0%	(*n* = 5) 19.2%		(*n* = 2) 12.5%
	AG	(*n* = 26) 44.05%	(*n* = 4) 23.5%	(*n* = 14) 53.8%		(*n* = 8) 50%
	GG	(*n* = 26) 44.05%	(*n* = 13) 76.5%	(*n* = 7) 27%		(*n* = 6) 37.5%
	A	0.339	0.118	0.462		0.375
	G	0.661	0,882	0.538		0.625
	Statistical analysis	Poor vs. normo	Normo vs. hyper	Poor vs. hyper		
		0.139	1.000	0.103	*P* value	AA vs. AG+GG
		1.810	1.032	2.308	OR	
		1.59–2.409	0.210–5.058	1.533–3.475	95% CI	
		0.080	0.322	**0.002**	*P* value	AA+AG vs.GG
		0.113	2.111	0.239	OR	
		0.027–.467	0.567–7.855	0.054–1.066	95% CI	
		**0.001**	0.436	**0.015**	*P* value	A vs. G
		0.156	1.429	0.222	OR	
		0.048–0.505	0.581–3.513	0.063–0.787	95% CI	
*FSHR* rs6166		*N* = 60	*N* = 17	*N* = 26		*N* = 17
	AA	(*n* = 20) 33.3%	(*n* = 7) 41.2%	(*n* = 9) 34.6%		(*n* = 4) 23.5%
	AG	(*n* = 28) 46.7%	(*n* = 7) 41.2%	(*n* = 13) 50%		(*n* = 8) 47.1%
	GG	(*n* = 12) 20%	(*n* = 3) 17.6%	(*n* = 4) 15.4%		(*n* = 5) 29.4%
	A	0.567	0.618	0.596		0.471
	G	0.433	0.382	0.404		0.529
	Statistical analysis	Poor vs normo	Normo vs hyper	Poor vs hyper		
		0.528	0.740	0.465	*P* value	AA vs AG+GG
		1.575	1.333	2.100	OR	
		0.440–5.638	0.327–5.434	0.474–9.297	95% CI	
		1.000	0.465	0.438	*P* value	AA+AG vs.GG
		1.111	1.909	2.121	OR	
		0.228–5.411	0.453–8.044	0.414–10.87	95% CI	
		0.582	0.428	0.225	*P* value	A vs. G
		1.282	1.429	1.831	OR	
		0.530–3.005	0.590–3.459	0.687–4.878	95% CI	
*ESR1* rs2234693		*N* = 60	*N* = 17	*N* = 26		*N* = 17
	CC	(*n* = 11) 18.3%	(*n* = 4) 23.5%	(*n* = 4) 15.4%		(*n* = 3) 17.6%
	CT	(*n* = 34) 56.7%	(*n* = 8) 47.1%	(*n* = 18) 69.2%		(*n* = 8) 47.1%
	TT	(*n* = 15) 25%	(*n* = 5) 29.4%	(*n* = 4) 15.4%		(*n* = 6) 35.3%
	C	0.467	0.471	0.500		0.412
	T	0.533	0.529	0.500		0.588
	Statistical analysis	Poor vs normo	Normo vs hyper	Poor vs hyper		
		0.92	1.000	1.000	*P* value	CC vs. CT+TT
		1.692	0.788	1.333	OR	
		0.361–7.943	0.152–4.088	0.248–7.174	95% CI	
		0.481	0.465	1.000	*P* value	CC + CT vs. TT
		0.571	1.909	1.01	OR	
		0.137–2.384	0.453–8.044	0.247–4.817	95% CI	
		0.926	0.699	0.787	*P* value	C vs. T
		0.960	1.190	1.143	OR	
		0.404–2.282	0.491–2.885	3.015–0.433	95% CI	

Notes

*P* is from Fisher exact test

Analyzed correlations between SNP genotypes and clinical characteristics are included in Table 4.

**Table 4:** Statistically significant associations between selected SNPs and patients’ baseline hormonal values and rFSH dose used for ovarian hyperstimulation

Gene	SNP	Characteristic	Genotype	Median (interquartile range)	*P* value	
*AMHR*	rs3741664	rFSH (IU)	AG	1420 (338)	**0.028**	AG vs. GG
			GG	2025 (1350)		
*FSHR*	rs1394205	AMH (ng/mL)	AA	6.80 (8.92)	**0.016**	AA+AG vs.GG
			AG	2.83 (5.33)		
			GG	1.36 (3.01)		
		rFSH (IU)/oocyte	AA	147.92 (268.21)	**0.036**	AA+AG vs.GG
			AG	125.00 (108.04)		
			GG	235.71 (571.88)		
*FSHR*	rs6166	bFSH (mIU/mL)	AA	5.75 (2.83)	**0.043**	AA vs. AG+GG
			AG	5.25 (2.94)		
			GG	4.60 (1.50)		
*ESR1*	rs2234693	Estradiol on hCG day (pmol/L)	CC	1.980 (3.44)	**0.038**	CC vs CT+TT
			CT	4.625 (5.36)		
			TT	3.060 (9.18)		

Notes: *P* < 0.05 was considered statistically significant. Statistically significant values are written in bold

*bFSH* basal follicle-stimulating hormone, *AMH* basal anti-Müllerian hormone, *hCG* human chorionic gonadotropin, *rFSH* recombinant follicle-stimulating hormone, *IU* international units

*P* is from Mann-Whitney test

SNPs rs3741664 in *AMHR* gene as well as rs1394205 and rs6166 in *FSHR* gene were positively associated with serum AMH, FSH, and rFSH dose used. Selected SNPs did not show any association with oocyte number.

Patients with GG genotype of SNP rs1394205 in *FSHR* gene had lower measured serum AMH level (*P* = 0.016), and required higher rFSH dose per oocyte (*P* = 0.036) than patients with AA or AG genotype.

Patients with AA genotype of SNP rs6166 in *FSHR* gene had higher level of measured basal serum FSH compared to those with AG or GG genotypes (*P* = 0.043).

Women with GG genotype of SNP rs3741664 in *AMHR* gene required higher rFSH dose in COS in comparison with patients carrying genotype AG (*P* = 0.028).

## Discussion

Ovarian stimulation in MAR involves the use of exogenous gonadotropins, which can result in an excessive response including OHSS, or inadequate response leading even to the cancelation of the IVF cycle. This significant variability in response has been the focus of many pharmacogenetic studies, which have analyzed the relationship between selected SNPs in candidate hormonal receptor genes involved in folliculogenesis and ovarian response to COH [6–8, 11, 12]. Among them, SNPs rs10407022 in gene *AMH*, rs3741664 in gene *AMHR*, rs1394205 and rs6166 in gene *FSHR*, and rs2234693 in gene *ESR1* were the largely studied genes to date. Each of the mentioned studies analyzed only single SNP or only several SNPs in the same gene. In our study we were focused on several genes and their polymorphisms of various hormonal receptors involved in folliculogenesis. By finding association between different polymorphisms of selected genes, their genotypes and parameters characterizing ovarian response to COH, new genetic biomarkers for prediction of ovarian stimulation could be identified.

Due to important roles of FSH in follicular growth and ovarian steroidogenesis in females, mutations in *FSHR* gene could affect reproductive ability [7]. Two polymorphisms, rs6165 and rs6166 in *FSHR* gene are in almost complete linkage disequilibrium [27]. This is why most of the studies were focused only on SNP rs6166, as genotyping of either of them permits genotype inference of the other.

In the present study we investigated the association of rs6166 *FSHR* polymorphism with the clinical and endocrinologic parameters of study group patients. The results showed the highest frequency distribution of the AG genotype and are consistent with high AG genotype distribution found also in other ethnic groups [6–8, 11, 12].

It has been reported that basal FSH (bFSH) levels differ significantly among the rs6166 genotype variants with carriers of the GG genotype, having slightly higher bFSH levels and requiring a significantly higher gonadotropin dose to induce ovulation [5, 6, 11, 28–31]. In the present study bFSH was different among various genotype variants. Similar to other studies, patients with AA genotype had higher bFSH compared to women with AG and GG genotypes in one group (*P* = 0.043) [8, 12, 13, 32].

Yan et al. [33] reported that subjects with AA genotypes had higher basal FSH levels, and that these genotypes were associated with an increased risk of poor response. Their data suggested that personalized FSH therapy may be applied according to patient’s genetic background in clinical settings. Also, results of allele frequency analysis showed higher frequency of allele A in poor responders. From all these studies, it can be concluded that the A allele is associated with poor ovarian response to gonadotropin therapy. We could consider that patients with the genotype AA were less sensitive to FSH, because they have increased bFSH and hence may require a higher dose of rFSH to normal follicle development.

Lindgren et al. [34] suggested that combination of SNPs from more hormone receptors involved in folliculogenesis could give more reliable COS outcomes prediction value. In their study performed on IVF, women of a Caucasian origin, *FSHR* rs6166 and *LHCGR* rs2293275 SNPs alone were not associated with increased live birth rate. But, when they combined receptors, they found that women homozygous for serine in both *FSHR* rs6166 and *LHCGR* rs2293275 had approximately 40% higher live birth rate compared to those with other receptor variants.

In our study SNP rs1394205 in *FSHR* gene was also analyzed. The data revealed that women with GG genotype at the rs1394205 position were classified more often as poor responders compared to women with AA genotype. They needed a higher amount of exogenous FSH per oocyte retrieved compared to AA and AG genotype patients (*P* = 0.036). These results indicate that the SNP rs1394205 in *FSHR* gene may influence sensitivity of the FSHR to FSH.

SNP rs1394205 is located in the promoter region of *FSHR* gene and has been associated with altered transcriptional activity of the *FSHR* gene [35]. It is suggested that the reduced *FSHR* expression at the transcript level is in concurrence with the expression of *FSHR* at the protein level [36]. Chai et al. [37] report that the expression of *FSHR* gene, at both the mRNA and protein levels, is significantly different among the three groups (poor, normo, and hyper responders), with the lowest expression in the poor responders. They observe the highest dosage of rFSH and the higher levels of FSH in follicular fluid of poor responders. Because the secretion of FSH is in a negative feedback loop with the action of *FSHR*, the basal levels of FSH are often indicative of the function of *FSHR* [38], which suggests that increased administration of gonadotropin might elevate the local concentration of FSH and improve the oocyte development. The findings suggest that increasing the dose of rFSH does not improve oocyte development probably due to insufficiency of *FSHR* expression on granulosa cells [37].

Wunsch et al. [32] analyzed whether there are ethnic differences concerning the SNPs in the promoter region in DNA samples of 55 Indonesian women. Interestingly, a different distribution pattern was found compared with the Caucasian population. The distribution in German patients was as follows: GG (55.4%), AG (37.6%), and AA (6.9%), while the Indonesian women showed the following repartition: GG (29%), AG (49%), and AA (22%). Distribution of analyzed SNP genotypes in our study group was GG (44.05%), AG (44.05%), and AA (11.9%). In another study on Indian women, those with AA genotype of SNP rs1394205 in *FSHR* gene were compared with GG genotype women and it was revealed that the AA genotype required higher dose of exogenous FSH for ovarian hyperstimulation [36]. They concluded that AA genotype at position rs1394205 might be associated with poor ovarian response. Findings from their study consistently demonstrate a correlation of the AA genotype at rs1394205 position of the FSHR gene with poor ovarian response. Interestingly, when they compared the FSHR expression at protein level on the basis of genotypes at position rs1394205, they observed that subjects with the AA genotype expressed significantly lower amounts of receptor protein compared with the GG and GA genotypes. Their observation thus suggests that the reduced FSHR expression at the transcript level is in concurrence with the expression of FSHR at the protein level in subjects with the AA genotype. The findings of the study carried out in Iran are similar to those results [10]. These results are in contrast with ours. The reason for the differences is probably in the various distributions of genotypes within different ethnic populations.

In our previous study [39], we analyzed whether we can predict the response to ovarian stimulation using AMH blood level. It was concluded that AMH is an independent and an accurate predictor for poor response on gonadotrophin stimulation. In the present study, AMH values were different among genotype variants. Patients with GG genotype had lower AMH values compared to women with AA and AG genotype in one group (*P =* 0.016). It can be concluded that GG genotype is strongly linked with poor response on ovarian stimulation.

By analyzing SNPs rs2234693 and rs4986938 in *ESR1* gene, we did not find any difference between genotype variants, neither in distribution of different genotypes between poor- and hyper-responders, nor in the amount of recombinant FSH used for COH, and number of oocytes retrieved. We found a statistically significant difference between genotype of rs2234693 and E2 on hCG day. Patients with CC genotype had lower E2 on hCG day compared to those with CT and TT genotypes. The data revealed that women with CC genotype at the rs2234693 position were classified more often as poor responders compared to women with CT or TT genotype. De Mattos et al. [40] studied the same SNPs in ESR1 gene. For SNP rs2234693 they did not confirm any influence on ovarian response, while the patients with rs2234693 TT genotype needed a higher dose of rFSH. Their results agree with prior findings by Altmäe et al. [16], and Ayvaz et al. [17], who demonstrated better ovarian response in patients with the rs2234693 CC genotype. Similar results have been published by de Castro et al. [41], who found lower frequency of C allele of SNP rs2234693 in poor responders.

Analysis of our data revealed that the women with GG genotype of SNP rs3714664 in *AMHR* gene received a higher amount of exogenous FSH for ovarian stimulation compared to patients with AG genotype. However, we did not find any positive association between these genotypes and ovarian response to gonadotropins or concentrations of basal hormones (bFSH, AMH, and E2 on the day of hCG administration).

Also, the polymorphism rs10407022 of the *AMH* gene was not associated either with any of measured hormonal parameters, or with response to ovarian stimulation. This does not confirm the results of some other studies in which such association was found with poor response [6, 42] or with ovarian hyperstimulation syndrome [43, 44]. On the other hand, the present results agree with those of Kerkelä et al. [45] who found no association between *AMH* coding polymorphisms and OHSS.

We can conclude that the GG genotype of SNP rs1394205 in *FSHR* is strongly linked with poor response on ovarian stimulation. Our study and patient classification into three groups was mainly based on the number of oocytes retrieved. Although oocyte yield increases with increasing dose of FSH, availability of blastocysts is less influenced by the rFSH dose and AMH level [46], and so blastocyst quality may be a more meaningful criterion for patient classification in COS response groups. This has to be considered in subsequent studies. Moreover, some studies in patients with endometriosis [47] and women of advanced age [48] have shown the impact of DNA methylation to gene expression in granulosa cells. The extension of such analyses on selected *FSHR*, *AMHR*, *AMH*, and *ESR1* genes is needed to confirm whether or not these SNPs could serve as potential biomarkers for prediction of COS outcome.

## References

[1] Altmäe S, Hovatta O, Stavreus-Evers A, Salumets A (2011). Genetic predictors of controlled ovarian hyperstimulation: where do we stand today?. Hum Reprod Update.

[2] Boudjenah R, Molina-Gomes D, Torre A, Bergere M, Bailly M, Boitrelle F. Genetic polymorphisms influence the ovarian response to rFSH stimulation in patients undergoing in vitro fertilization programs with ICSI. PLoS One. 2012. 10.1371/journal.pone.0038700. 10.1371/journal.pone.0038700 PMC3372493 22701696

[3] Belen L, Ortiz JA, Llacer J and Bernabeu R. Pharmacogenetics of ovarian response. 2014. 10.2217/pgs.14.49 10.2217/pgs.14.49 24897293

[4] Minegishi T, Nakamura K, Takakura Y, Ibuki Y, Igarashi M (1991). Cloning and sequencing of human FSH receptor cDNA. Biochem Biophys Res Commun.

[5] Desai SS, Roy BS, Mahale SD (2013). Mutations and polymorphisms in FSH receptor: functional implications in human reproduction. Reproduction.

[6] Mayorga MP, Gromoll J, Behre HM, Gassner C, Nieschlag E, Simoni M (2000). Ovarian response to follicle-stimulating hormone (FSH) stimulation depends on the FSH receptor genotype. J Clin Endocrinol Metab.

[7] Sudo S, Kudo M, Wada S, Sato O, Hsueh AJ, Fujimoto S (2002). Genetic and functional analyses of polymorphisms in the human FSH receptor gene. Mol Human Reprod.

[8] Kuijper EAM, Blankenstein MA, Luttikhof LJ, Roek SJM, Overbeek A, Hompes PG, Twisk JWR, Lambalk CB (2011). Frequency distribution of polymorphisms in the FSH receptor gene in infertility patients of different ethnicity. Reprod BioMed Online.

[9] Gashi Z, Elezaj S, Zeqiraj A, Grabanica D, Gashi F (2016). Follicle-stimulating hormone receptor gene polymorphism in Albanian women. Arch Med Sci Civil Dis.

[10] Bonyadi K, Damavandi E, Choobineh H, Kabuli M, Agha-Hosseini M, Ghadami M (2017). Association of FSH receptor promoter’s polymorphisms with IVF-failure in Iranian women. Int J Reprod Contracept Obstet Gynecol.

[11] Jun JK, Yoon JS, Ku SY (2006). Follicle-stimulating hormone receptor gene polymorphism and ovarian responses to controlled ovarian hyperstimulation for IVF-ET. J Human Genet.

[12] Mohiyiddeen L, Newman WG, Cerra C, McBurney H, Mulugeta B, Roberts SA, Nardo LG (2013). A common Asn680Ser polymorphism in the follicle-stimulating hormone receptor gene is not associated with ovarian response to gonadotropin stimulation in patients undergoing in vitro fertilization. Fertil Steril.

[13] Wunsch A, Ahda Y, Banaz-Yas F, Sonntag B, Nieschlag E, Simoni M (2005). Single-nucleotide polymorphisms in the promoter region influence the expression of the human follicle stimulating hormone receptor. Fertil Steril.

[14] Goldenberg RL, Vaitukaitis JL, Ross GT (1972). Estrogen and follicle stimulation hormone interactions on follicle growth in rats. Endocrinology.

[15] Georgiou I, Konstantelli M, Syrrou M, Messinis IE, Lolis DE (1997). Oestrogen receptor gene polymorphisms and ovarian stimulation for in-vitro fertilization. Hum Reprod.

[16] Sundarrajan C, Liao W, Roy AC, Ng SC (1999). Association of oestrogen receptor gene polymorphisms with outcome of ovarian stimulation in patients undergoing IVF. Mol Hum Reprod.

[17] Altmäe S, Haller K, Peters M, Hovatta O, Stavreus-Evers A, Karro H, Metspalu A, Salumets A (2007). Allelic estrogen receptor 1 (ESR1) gene variants predict the outcome of ovarian stimulation in in vitro fertilization. Mol Hum Reprod.

[18] Ayvaz OU, Ekmekçi A, Baltaci V, Onen HI, Unsal E (2009). Evaluation of in vitro fertilization parameters and estrogen receptor alpha gene polymorphisms for women with unexplained infertility. J Assist Reprod Genet.

[19] He M, Shu J, Huang X, Tang H (2015). Association between estrogen receptor gene (ESR1) PvuII (T/C) and XbaI (A/G) polymorphisms and premature ovarian failure risk: evidence from a meta-analysis. J Assist Reprod Genet.

[20] Rigon C, Andrisani A, Forzan M, D'Antona D, Bruson A, Cosmi E, Ambrosini G, Tiboni GM, Clementi M (2010). Association study of AMH and AMHRII polymorphisms with unexplained infertility. Fertil Steril.

[21] Weenen C, Laven JS, Von Bergh AR, Cranfield M, Groome NP, Visser JA (2004). Anti-Müllerian hormone expression pattern in the human ovary: potential implications for initial and cyclic follicle recruitment. Mol Hum Reprod.

[22] Visser JA, Themmen APN (2005). Anti-Mullerian hormone and folliculogenesis. Mol Cell Endocrinol.

[23] Durlinger AL, Gruijters MJG, Kramer P, Karels B, Ingraham HA, Nachtigal MW (2002). Anti-Mullerian hormone inhibits initiation of primordial follicle growth in the mouse ovary. Endocrinology.

[24] Durlinger AL, Kramer P, Karels B, de Jong FH, Uilenbroek JTJ, Grootegoed A (1999). Control of primordial follicle recruitment by anti-Müllerian hormone in the mouse ovary. Endocrinology.

[25] Durlinger AL, Gruijters MJG, Kramer P, Karels B, Matzuk MM, Rose UM (2001). Anti-Müllerian hormone attenuates the effects of FSH on follicle development in the mouse ovary. Endocrinology.

[26] Kevenaar ME, Themmen AP, Laven JS, Sonntag B, Fong S, de Jong F (2007). Anti-Mullerian hormone and anti-Mullerian hormone type II receptor polymorphisms are associated with follicular phase oestradiol levels in normo-ovulatory women. Hum Reprod.

[27] Simoni M, Livio C (2014). Mechanisms in endocrinology: genetics of FSH action - a 2014-and-beyond view. Eur J Endocrinol.

[28] Falconer H, Andersson E, Aanesen A, Fried G (2005). Follicle stimulating hormone receptor polymorphisms in a population of infertile women. Acta Obstet Gynecol Scand.

[29] Loutradis D, Patsoula E, Minas V, Koussidis GA, Antsaklis A, Michalas S, Makrigiannakis A (2006). FSH receptor gene polymorphisms have a role for different ovarian response to stimulation in patients entering IVF/ICSI-ET programs. J Assist Reprod Genet.

[30] Yao Y, Ma CH, Tang HL, Hu YF (2011). Influence of follicle-stimulating hormone receptor (FSHR) Ser680Asn polymorphism on ovarian function and in-vitro fertilization outcome: a meta-analysis. Mol Genet Metabol.

[31] Huang X, Li L, Hong L (2015). The Ser680Asn polymorphism in the follicle-stimulating hormone receptor gene is associated with the ovarian response in controlled ovarian hyperstimulation. Clin Endocrinol.

[32] Sheikhha MH, Eftekhar M, Kalantar SM (2011). Investigating the association between polymorphism of follicle-stimulating hormone receptor gene and ovarian response in controlled ovarian hyperstimulation. J Human Reprod Sci.

[33] Yan Y, Gong Z, Zhang L, Li Y, Li X, Zhu L, et al. Association of follicle-stimulating hormone receptor polymorphisms with ovarian response in Chinese women: a prospective clinical study. PLoS One. 2013. 10.1371/journal.pone.0078138. 10.1371/journal.pone.0078138 PMC3805513 24167601

[34] Lindgren I, Nenonen H, Henic E, Bungum L, Prahl A, Bungum A, et al. Gonadotropin receptor variants are linked to cumulative live birth rate after in vitro fertilization. J Assist Reprod Genet. 2018. 10.1007/s10815-018-1318-y. 10.1007/s10815-018-1318-y PMC6338601 30232643

[35] Nakayama T, Kuroi N, Sano M, Tabara Y, Katsuya T, Ogihara T, Makita Y, Hata A, Yamada M, Takahashi N, Hirawa N, Umemura S, Miki T, Soma M (2006). Mutation of the follicle-stimulating hormone receptor gene 5′-untranslated region associated with female hypertension. Hypertension.

[36] Desai SS, Achrekar SK, Pathak BR, Desai SK, Mangoli VS, Mangoli RV, Mahale SD (2011). Follicle-stimulating hormone receptor polymorphism (g-29a) is associated with altered level of receptor expression in granulosa cells. J Clin Endocrinol Metab.

[37] Cai J, Lou H, Dong M, Lu XE, Zhu YM, Gao HJ (2007). Poor ovarian response to gonadotropin stimulation is associated with low expression of follicle-stimulating hormone receptor in granulosa cells. Fertil Steril.

[38] Gerasimova T, Thanasoula MN, Zattas D, Seli E, Sakkas D, Lalioti MD (2010). Identification and in vitro characterization of follicle stimulating hormone (FSH) receptor variants associated with abnormal ovarian response to FSH. J Clin Endocrinol Metab.

[39] Knez J, Kovačič B, Medved M, Vlaisavljević V. What is the value of anti-Müllerian hormone in predicting the response to ovarian stimulation with GnRH agonist and antagonist protocols? Reprod Biol Endocrinol. 2015. 10.1186/s12958-015-0049-5. 10.1186/s12958-015-0049-5 PMC4470079 26059906

[40] de Mattos CS, Trevisan CM, Peluso C, Adami F, Cordts EB, Christofolini DM, Barbosa CP, Bianco B (2014). ESR1 and ESR2 gene polymorphisms are associated with human reproduction outcomes in Brazilian women. J Ovarian Res.

[41] de Castro F, Morán FJ, Montoro L, Galán JJ, Pérez- Hernández D, ES-C P (2004). Human controlled ovarian hyperstimulation outcome is a polygenic trait. Pharmacogenet Genom.

[42] Behre HM, Hermann M, Greb R, Mempel A, Sonntag B, Kiesel L (2005). Significance of a common single nucleotide polymorphism in exon 10 of the follicle-stimulating hormone (FSH) receptor gene for the ovarian response to FSH: a pharmacogenetic approach to controlled ovarian hyperstimulation. Pharmacogenet Genom..

[43] Daelemans C, Smits G, de Maertelaer V, Costagliola S, Englert Y, Vassart G, Delbaere A (2004). Prediction of severity of symptoms in iatrogenic ovarian hyperstimulation syndrome by follicle-stimulating hormone receptor Ser680Asn polymorphism. J Clin Endocrinol Metab.

[44] Achrekar SK, Modi DN, Desai SK, Mangoli VS, Mangoli RV, Mahale SD (2009). Poor ovarian response to gonadotrophin stimulation is associated with FSH receptor polymorphism. Reprod BioMed Online.

[45] Kerkelä E, Skottman H, Friden B, Bjuresten K, Kere J, Hovatta O (2007). Exclusion of coding-region mutations in luteinizing hormone and follicle-stimulating hormone receptor genes as the cause of ovarian hyperstimulation syndrome. Fertil Steril.

[46] Arce JC, Andersen AN, Fernández-Sánchez M, Visnova H, Bosch E, García-Velasco JA (2014). Ovarian response to recombinant human follicle-stimulating hormone: a randomized, antiMüllerian hormone-stratified, dose-response trial in women undergoing in vitro fertilization/intracytoplasmic sperm injection. Fertil Steril.

[47] Hayashi M, Yamashita Y, Hayashi A, Yoshida Y, Kawabe S, Hayashi M (2012). Expression and epigenetic change of the AR and FSHR genes in the granulosa cells of endometriosis patients. Genet Epigenet.

[48] Yu B, Russanova VR, Gravina S, Hartley S, Mullikin JC, Ignezweski A (2015). DNA methylome and transcriptome sequencing in human ovarian granulosa cells links age-related changes in gene expression to gene body methylation and 3′-end GC density. Oncotarget.

